# Recognition of Antifungal-Resistant Dermatophytosis by Infectious Diseases Specialists, United States

**DOI:** 10.3201/eid3009.240118

**Published:** 2024-09

**Authors:** Jeremy A.W. Gold, Kaitlin Benedict, Shawn R. Lockhart, Caitlyn Lutfy, Meghan Lyman, Dallas J. Smith, Philip M. Polgreen, Susan E. Beekmann

**Affiliations:** Centers for Disease Control and Prevention, Atlanta, Georgia, USA (J.A.W. Gold, K. Benedict, S.R. Lockhart, C. Lutfy, M. Lyman, D.J. Smith);; University of Iowa Carver College of Medicine, Iowa City, Iowa, USA (P.M. Polgreen, S.E. Beekmann)

**Keywords:** dermatophytosis, dermatomycoses, tinea, Trichophyton, Trichophyton indotineae, Trichophyton rubrum, drug resistance, fungi, antifungal agents, terbinafine, itraconazole, antimicrobial stewardship, United States, ringworm

## Abstract

Antifungal-resistant dermatophyte infections have recently emerged as a global public health concern. A survey of US infectious diseases specialists found that only 65% had heard of this issue and just 39% knew how to obtain testing to determine resistance. Increased clinician awareness and access to testing for antifungal-resistant dermatophytosis are needed.

Dermatophytosis (i.e., ringworm, tinea) is a common superficial fungal skin infection caused by dermatophyte molds (*1*). In the past decade, widespread outbreaks of antifungal-resistant dermatophytosis have occurred in South Asia because of the spread of *Trichophyton indotineae* fungus, which causes extensive pruritic plaques on the trunk, extremities, groin, and face in immunocompetent persons (*2*–*4*). Unlike typical dermatophyte skin infections, those involving *T*. *indotineae* fungus often do not resolve with over-the-counter topical antifungals or oral terbinafine (first-line systemic therapy) (*2*,*3*).

*T*. *indotineae* fungus has been detected in at least 11 US states, and recalcitrant dermatophytosis caused by antifungal-resistant *T. rubrum* fungus also has been reported (*2*,*5*,*6*). Affected patients have experienced diagnostic delays and received inappropriate therapies, including topical corticosteroids, which can worsen dermatophytosis (*2*). Antifungal-resistant dermatophytosis cases in the United States probably are underrecognized because this condition is not reportable to public health authorities in any state (https://www.cdc.gov/fungal/php/case-reporting) and most superficial fungal infections are not confirmed with diagnostic testing (*7*). Further, laboratory capacity to identify dermatophyte species and perform antifungal-susceptibility testing (AFST) may be limited, given that definitive identification of certain species, such as *T*. *indotineae,* requires advanced molecular techniques (e.g., using internal transcribed spacer region sequencing) and most clinical laboratories do not perform AFST for molds (*2*,*8*).

Antifungal-resistant dermatophytosis cases might require infectious diseases (ID) clinician consultation both to manage antifungal use and because of the potential effect of these infections on highly immunocompromised patients. Understanding ID clinicians’ level of awareness regarding antifungal-resistant dermatophytosis and access to laboratory testing could improve strategies to increase disease recognition and facilitate early appropriate treatment. Therefore, we surveyed ID clinicians by using the Emerging Infections Network (EIN) (https://ein.idsociety.org), a sentinel network of ID physicians and other ID specialists.

During December 2023, EIN distributed a survey link to ≈3,000 member subscribers on 3 separate occasions ≈1 week apart. EIN queries are designated as non–human subjects research by the institutional review board of the University of Iowa.

In total, we received 158 responses (Table). The most common practice setting was university hospital (47%), followed by community hospital (16%) and nonuniversity teaching hospital (12%). Most respondents were adult ID physicians (80%), followed by pediatric ID physicians (12%), pharmacists (4%), nurse practitioners, physician assistants or physician associates (1%), or other (3%). Overall, 103 (65%) respondents had heard of antifungal-resistant dermatophytosis before receiving the survey; among those, most (58%) had heard of it through previous EIN listserv emails. Seventeen percent of respondents reported seeing or consulting on a patient who had dermatophytosis that was potentially resistant to treatment or concerning for resistance within the previous year.

**Table:** Survey regarding 158 infectious diseases clinicians’ awareness of antifungal-resistant dermatophyte infections and access to laboratory testing, United States, 2023

Survey query	No. (%) respondents
Primary setting of clinical practice	
University hospital	75 (47)
Community hospital	26 (16)
Non-university teaching hospital	19 (12)
Veterans Affairs hospital or Department of Defense	13 (8)
Outpatient setting only	8 (5)
Free-standing children’s hospital	5 (3)
City, county, or public hospital	4 (3)
Other	4 (3)
Question not answered	4 (3)
Type of clinical practice	
Adult infectious diseases physician	126 (80)
Pediatric infectious diseases physician	19 (12)
Pharmacist	6 (4)
Nurse practitioner, physician assistant, or physician associate	2 (1)
Other	4 (3)
Question not answered	1 (1)
How did you hear about the issue of antifungal-resistant dermatophyte infections? (select all that apply)	
I had not previously heard of this issue	55 (35)
Answers among those who had heard of this issue, n = 103	
Emerging Infections Network emails	60 (58)
CDC webpage or *Morbidity and Mortality Weekly Report*	46 (45)
Scientific publication	28 (27)
News reports	18 (17)
From clinical colleagues	18 (17)
Clinician education website (e.g., Doximity and Medscape)	6 (6)
Point-of-care medical resource (e.g., UpToDate)	4 (4)
Social media	2 (2)
Other	6 (6)
In the past year, I have seen or consulted on a patient with a dermatophyte infection that was potentially resistant to treatment or concerning for resistance	
Yes	27 (17)
No	119 (75)
Unknown	12 (8)
If I saw a patient with a potentially resistant dermatophyte infection, I would be able to obtain laboratory testing to determine the species	
Yes	83 (53)
No	26 (16)
Unknown	49 (31)
I would be able to obtain testing to determine if a dermatophyte is resistant to antifungals	
Yes	62 (39)
No	25 (16)
Unknown	71 (45)

Approximately half (47%) of respondents reported that if they saw a patient with potentially resistant dermatophytosis, they either would not be able to obtain laboratory testing to determine the species (16%) or were unsure whether they could obtain such testing (31%) (Table). Likewise, most respondents (61%) reported they would either not be able to obtain testing to determine if a dermatophyte was resistant to antifungals (16%) or were unsure whether they could obtain such testing (45%).

In summary, our survey found that approximately one third (35%) of ID clinicians had not heard of antifungal-resistant dermatophytosis. Only 53% of respondents reported knowing how to obtain access to dermatophyte speciation testing, and even fewer (39%) reported knowing how to obtain testing for dermatophyte resistance (Table). Those findings probably reflect the relatively recent recognition of these infections in the United States and the fact that testing to identify *T. indotineae* and other resistant dermatophyte species is limited to select US mycology reference centers and public health laboratories (*2*,*8*). 

Dermatophyte species identification and AFST are critical to guiding antifungal treatment decisions for patients with potentially resistant dermatophytosis and for monitoring population-level trends in resistance profiles to inform treatment guidelines (*8*). National guidelines for treating antifungal-resistant dermatophytosis are lacking, but the azole antifungal itraconazole has been used successfully for patients with dermatophytosis caused by *T. indotineae* and terbinafine-resistant *T. rubrum* fungi (*2*,*9*). However, clinicians should be aware of challenges with itraconazole, including pharmacokinetic variability (e.g., absorption), insurance coverage, drug–drug interactions, need for prolonged treatment (e.g., >6 weeks), and reports of emerging itraconazole-resistant *T. indotineae* and *T. rubrum* fungi (*2*,*6*,*9*,*10*).

One limitation of our survey is its low response rate and nonrepresentative nature. Furthermore, the survey might overestimate clinician awareness of antifungal-resistant dermatophytosis and laboratory testing access if clinicians experienced in this topic were likelier to respond. Despite those limitations, our survey highlights a need for increased awareness of antifungal-resistant dermatophytosis and increased laboratory capacity to identify and perform susceptibility testing for dermatophytes to address this emerging public health concern.

Healthcare professionals can find information about recognizing, diagnosing, treating, and reporting emerging dermatophyte infections online (https://www.aad.org/member/clinical-quality/clinical-care/emerging-diseases/dermatophytes), including information about laboratories that can perform testing (https://www.aad.org/member/clinical-quality/clinical-care/emerging-diseases/dermatophytes/recognizing-trichophyton-indotineae#testing). Those websites were developed as a collaboration between the Centers for Disease Control and Prevention and the American Academy of Dermatology’s Emerging Diseases Task Force. 
